# Radiological Outcomes of Femoral Head Resection in Patients with Cerebral Palsy: A Retrospective Comparative Study of Two Surgical Procedures

**DOI:** 10.3390/children8121105

**Published:** 2021-12-01

**Authors:** Axel Horsch, Finja Hahne, Maher Ghandour, Hadrian Platzer, Merkur Alimusaj, Cornelia Putz

**Affiliations:** Department of Orthopedics and Trauma Surgery, Heidelberg University Hospital, Schlierbacher Landstraße 200a, 69118 Heidelberg, Germany; finja.hahne@med.uni-heidelberg.de (F.H.); mghandourmd@gmail.com (M.G.); hadrian.platzer@med.uni-heidelberg.de (H.P.); merkur.alimusaj@med.uni-heidelberg.de (M.A.); cornelia.putz@med.uni-heidelberg.de (C.P.)

**Keywords:** Cerebral palsy, femoral head resection, femoral head cap plastic surgery, FCP, FHR

## Abstract

Background: We conducted this study to compare postoperative radiological outcomes of two surgical procedures (femoral head resection (FHR) and femoral head cap plastic surgery (FCP)) in patients with CP and hip dislocation. Methods: CP patients with Gross Motor Function Classification Score (GMFCS) IV or V, who underwent either FHR or FCP between 2007 and 2018 at Heidelberg University Hospital in Germany, were included. Most participants underwent postoperative traction in an attempt to prevent telescoping. Besides the above-mentioned objectives, we examined the association between telescoping and spasmolytic use, traction weight, and traction duration. Results: Thirty-eight CP patients were included, of whom 15 (25 hips) underwent FHR and 23 (30 hips) underwent FCP. Heterotopic ossification (grades I, II, and III) occurred in 80% and 83.3% of patients in the FHR and FCP groups, respectively. Telescoping occurred in 18.68 and 31.99% of patients in the FHR and FCP groups, respectively (*p* = 0.999). Other complications were similar between both groups. Conclusions: The postoperative outcomes of FHR and FCP are similar in terms of telescoping, heterotopic ossification, and complications. Although telescoping was encountered more in the FCP group, no significant difference from the FHR group was found. We noted that the weight of traction could reduce the development of telescoping.

## 1. Introduction

Cerebral palsy (CP) is characterized by a fixed lesion that affects the neurological system during development. Based on recent statistics, CP is known to affect 2.4–2.7 per thousand live births [1]. The presentation of CP differs widely based on the affected area of the brain. It is also classified into different subtypes, the most important of which is the spastic type, which is characterized by abnormal movements in the developing musculoskeletal system [2].

Pathologic hip conditions, such as subluxation or dislocation, are of great concern in patients with CP due to a permanent spasticity in several key muscles [1]. The hip flexors and hip adductors are mainly responsible for hip subluxation during growth. These conditions also correspond with individual’s Gross Motor Function Classification System (GMFCS) grade. It is reported that around 0.6% of patients with CP and GMFCS grade I experience hip pathologies [2], while 70% of patients with GMFCS grade IV experience these complications [3,4]. Complete hip dislocations are commonly encountered in non-ambulatory CP patients with GMFCS grade IV and V, and this can be quite problematic if pain is experienced or when balance, posture of hygiene becomes affected.

Even though the presence of one of these complications in such patients does not necessitate immediate surgical interventions, available evidence indicates that these complications are the most frequent causes for pain in CP patients, with a rate of 27% in non-ambulatory individuals [5]. Other reports highlight that around 15% to 57% of the dislocated hips of non-ambulatory patients eventually cause pain. In addition, a large proportion of affected patients also suffer from scoliosis, which leads to a pelvic tilt with a change in the acetabular entrance plane, which in turn promotes luxation [6,7].

The management of this patient population includes both reconstructive surgery, which is aimed to center the dislocated femoral head into the acetabulum, and salvage surgery, which is performed to reduce associated pain and/or functional deficits (e.g., sitting problems). The presence or absence of dislocation is the decisive factor about which of these two methods will be performed, taking into account whether or not there is loss of the spherical contour of the femoral head [8].

There are many options for salvage management of dislocated hips in CP patients, including femoral head resection (FHR) either with or without capping, which is known as femoral head cap plastic surgery (FCP), proximal femoral valgus osteotomy, hip arthrodesis, and prosthetic hip arthroplasty [9]. Noteworthy, pain and muscular spasm are frequent postoperative complaints during the early postoperative period, particularly before the benefits of FCP and FHR can be witnessed. Thus, a number of management strategies can be used to control these symptoms, including the use of analgesics, anxiolytics, or skin traction [10].

To date, there is no conclusive evidence to determine which option is superior compared to the others in terms of efficacy and postoperative complications in CP patients due to the lack of a comparison group, the small number of included patients, and the short follow-up periods. Therefore, we conducted this retrospective investigation to compare outcomes between FHR and FCP in terms of telescoping, heterotopic ossification, and long-term complications.

## 2. Materials and Methods

### 2.1. Study Design and Eligibility Criteria

**Figure 1:**
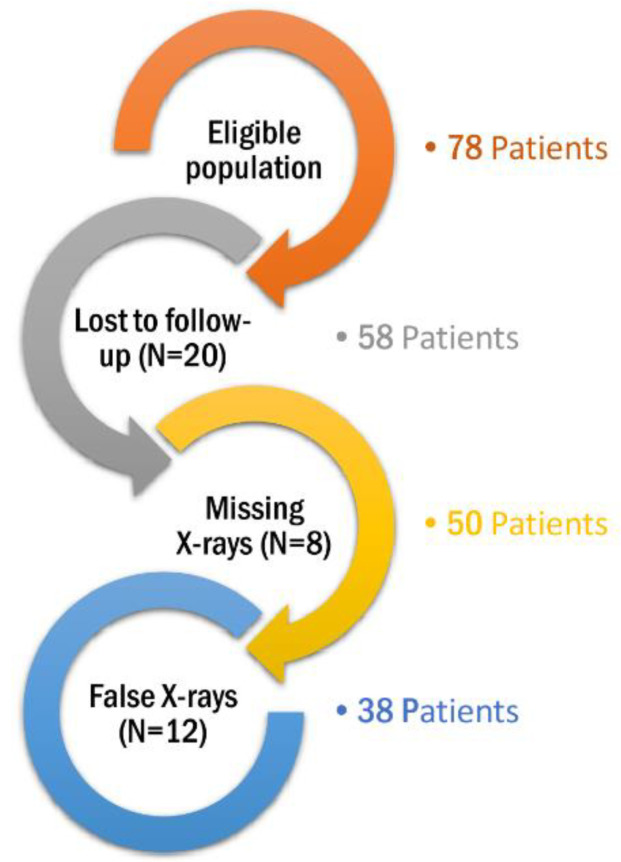
A flow chart summarizing the recruitment process of CP patients.

This retrospective cohort study was conducted among CP patients with hip dislocation who underwent either FHR or FCP during the period from 3 January 2007 to 13 December 2018 at the Heidelberg University Hospital, Department of Orthopedics and Trauma Surgery; Heidelberg, Germany. The inclusion criteria included patients with CP who underwent either FHR or FCP at our center during the study period and were followed-up after the selected operation for at least 2 years. We only included patients who had a significant functional disability representing a Gross Motor Function Classification System (GMFCS) IV (walking for short distances and requiring physical assistance) or V (having limited mobility and using wheelchairs) [11]. On the other hand, patients were excluded for the following reasons: (1) patients with paresis due to progressive neurological diseases, (2) the absence or non-clarity of radiographs during follow-up examinations, (3) losses to follow-up, (4) the lack of informed consent, and (5) the withdrawal from the study based on participants’ decisions or their parents or legal guardians. The process of patient selection is highlighted in (Figure 1).

The study protocol was approved by the Institutional Review Board (IRB)- Ethics Committee of the University Hospital Heidelberg prior to conducting the study (S-649/2019). All patient records were kept confidential, and eligible patients or their parents/guardians consented prior to participation in our study.

### 2.2. Study Procedures and Outcomes

We intended to determine the association between both operations (FHR and FCP) and the following outcomes: telescoping, heterotopic ossification, and postoperative complications. Radiographs at follow-up were used to identify both telescoping and heterotopic ossification. Radiographs of the last follow-up point were used in the analysis.

Telescoping is defined as the migration of the proximal part of the femur during follow-up. To adequately measure telescoping, postoperative radiographs of the operated hip were taken in an anterior-posterior (AP) position, and initial measurement of the maximum distance between the femur and the hip joint was taken. In the follow-up examinations, AP radiographs were also taken, and the aforementioned distance was measured again. To ensure the highest possible objectivity and reliability, measurements were always defined at the anatomical landmarks and in the same plane. For a thorough demonstration, the details of telescoping diagnosis are provided in (Supplementary Figure S1). At the hip joint itself, it was the tip of the hip’s articular tuberosity in the symphysis’s direction and, laterally, the acetabulum’s proximal lateral edge (line A). Exactly half of this line was measured in order to select the center of the joint as the starting point or origin (X). This was followed by another measurement: from the center of the joint to the highest point of the femur (line B). The femur’s migration, or telescoping, was expressed as a percentage to compensate for further inaccuracies. Thus, the femur’s complete migration back into the glenoid cavity corresponds to a telescoping of 100%. All procedures were carried out by the same surgeon.

**Figure 2:**
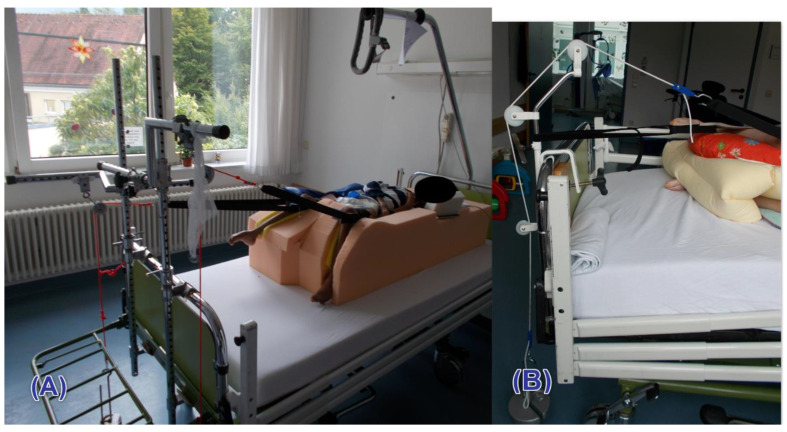
Illustration of postoperative traction for management of pain and muscle spasms. (**A**) traction in lateral decubitus; (**B**) traction in supine decubitus.

We further investigated the effect of spasmolytic therapy on telescoping, and therefore, we subdivided patients group according to spasmolytic use (Baclofen/Lioresal) into users and non-users. Moreover, we aimed to determine the association between the weight (<2 kg vs. ≥2 kg) and the duration of traction use (<6 months vs. ≥6 months) and telescoping. The method of traction is illustrated in (Figure 2A,B).

Heterotopic ossification was identified by radiographs at the last follow-up assessment, and it was graded based on the grading system that is proposed by Brooker et al. [12,13]. The grading of heterotopic ossification is provided in (Supplementary Table S1).

Complications following each procedure were classified based on the modified Clavien-Dindo-Sink classification system [14], and patients were assigned into different “complication groups” accordingly. The classification of complications is provided in (Supplementary Table S2).

### 2.3. Statistical Analysis

All statistical analyses were performed using Microsoft Excel 2016 and GraphPad Prism 8.4.3 software. Descriptive statistics in the form of numbers and percentages were used in categorical variables, while the mean and standard deviation (SD) were used in continuous variables. A *p*-value ≤ 0.05 was considered statistically significant.

The Chi-Square test was used to assess the difference in the incidence of heterotopic ossification and traction against femoral migration (telescoping) between the two groups. The Chi-Square test was also used to determine the influence of antispasmodics on telescoping outcome. Meanwhile, the unpaired t-test was performed for the traction weight and time.

## 3. Results

### 3.1. Participants’ Characteristics

Seventy-eight patients were eligible; however, we excluded 40 patients for the following reasons: missing follow-up periods (N = 20), missing x-rays (N = 8), and inadequate x-ray images (N = 12). Therefore, a total of 38 patients with CP were included in the final analysis. The demographic characteristics of included patients are provided in (Table 1). Fifteen patients (39.5%) underwent proximal FHR surgery, while 23 patients (60.5%) underwent FCP surgery.

In the FHR group, the majority of patients were males (60%), with a mean age of 25.93 years. In this group, 25 hips were operated upon, with 8 bilateral and 7 unilateral procedures. Most patients had severe functional limitations (GMFCS grade V = 86.7%), with a mean follow-up period of 32.25 months. Noteworthy, 5 patients had additional surgery: four in the FCP group (due to pain [N = 2], telescoping [N = 1], and cerclage-insufficiency [N = 1]) and one in the FHR group (due to pain).

Similarly, in the FCP group, most patients were males (60.9%), with a mean age of 26.74 years and a mean follow-up of 24.6 months. Also, the majority of patients (78.3%) who underwent FCP had GMFCS grade V at baseline. In this group, 30 hips were operated, with 8 bilateral and 15 unilateral procedures.

### 3.2. Procedural Outcomes

#### 3.2.1. Telescoping

Noteworthy, 4 patients in the overall population showed very noticeable telescoping values (considered as outliers). Meanwhile, telescoping, even in a limited degree, was present in all of the remaining patients.

In the FHR group (11 patients) an average telescoping of 18.68% (with a median of 12.86%) was noted who experienced telescoping postoperatively. On the other hand, the average telescoping in the FCP group (31.99%) was higher than that of the FHR group with a median telescoping of 14.33%). However, no significant difference was observed between both groups (*p* > 0.9999). Radiographic images showing cases with no telescoping and telescoping are provided in (Supplementary Figures S2 and S3). Of note, telescoping was associated with pain and posture and sitting problems. Pain was experienced by 24 patients during the preoperative period, while 11 patients experienced pain following the assigned procedure. Pain was treated with either Lioresal (3 patients), Botox injection (1 patient), or by adjusting the positioning of the machine (4 patients). On the other hand, 6 patients had sitting problems after the procedure that was treated with either adjusting the splint (2 patients), Lioresal (2 patients), or physiotherapy (2 patients).

#### 3.2.2. Spasmolytic Use and Telescoping

**Figure 3:**
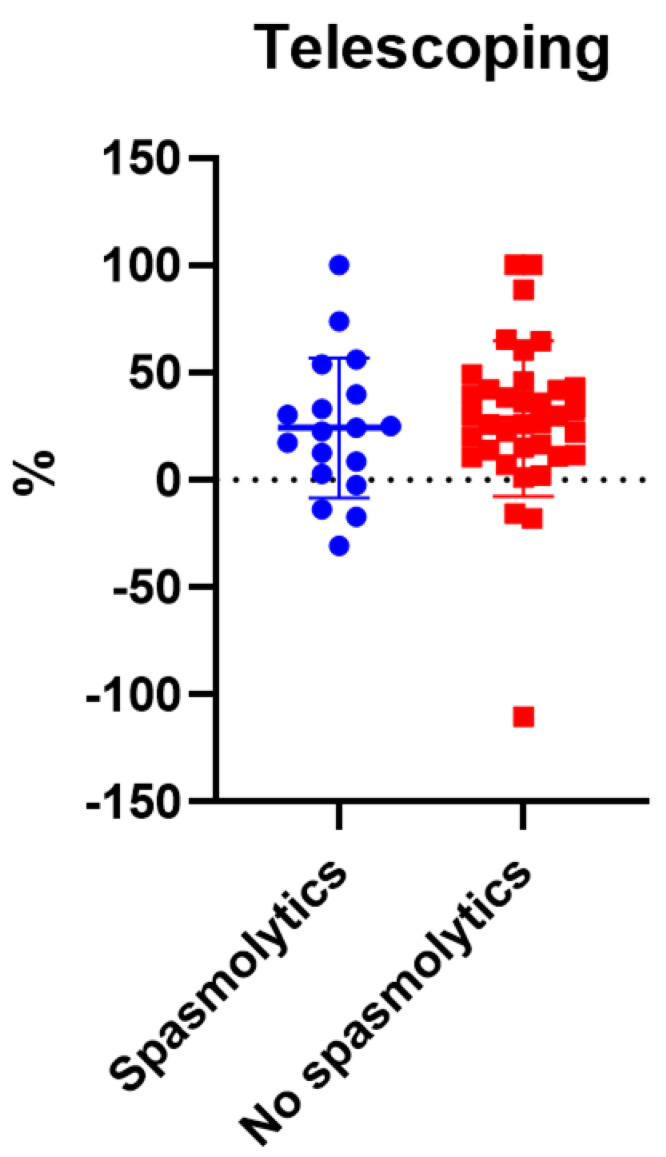
The correlation between spasmolytics use and telescoping.

We also investigated the correlation between spasmolytic use and telescoping in the overall patient population (both groups). A total of 18 patients used spasmolytics, and 19 patients did not use spasmolytics. Of note, the data of one patient was excluded because he was taking Baclofen (5 mg) preoperatively and he was not given Baclofen after the procedure. No statistically significant correlation was noted between spasmolytic use and postoperative telescoping (*p* = 0.4279) (Figure 3).

#### 3.2.3. Traction Weight and Telescoping

To prevent telescoping, traction was used in almost all patients (except for 4 cases). We distinguished between hips that had ≥2-kilo traction (*n* = 43) and hips that had <2-kilo traction (*n* = 3). Patients who did not use traction were excluded from this analysis. Patients in the ≥2 kilos traction group had significantly lower mean telescoping compared to the other group (27.02% vs. 100%; variance = 72.98%; *p* = 0.001) (Supplementary Figure S4).

#### 3.2.4. Traction Duration and Telescoping

We investigated the effect of traction duration (<6 months vs. ≥6 months) on telescoping. A total of 11 patients used traction for ≥6 months, while 8 patients used traction for <6 months. Patients without traction or with no data regarding the duration of traction use were excluded from this analysis. However, no statistically significant difference was noted (*p* = 0.9052).

### 3.3. Heterotopic Ossification

We determined the differences in heterotopic ossification between the FHR and FCP groups (Table 2). Most patients in the FHR and FCP groups had heterotopic ossification grade II (36% vs. 53.3%), respectively. Meanwhile, the occurrence of grade III was very minimal in both FHR and FCP groups (8% vs. 10%), respectively.

**Figure 4:**
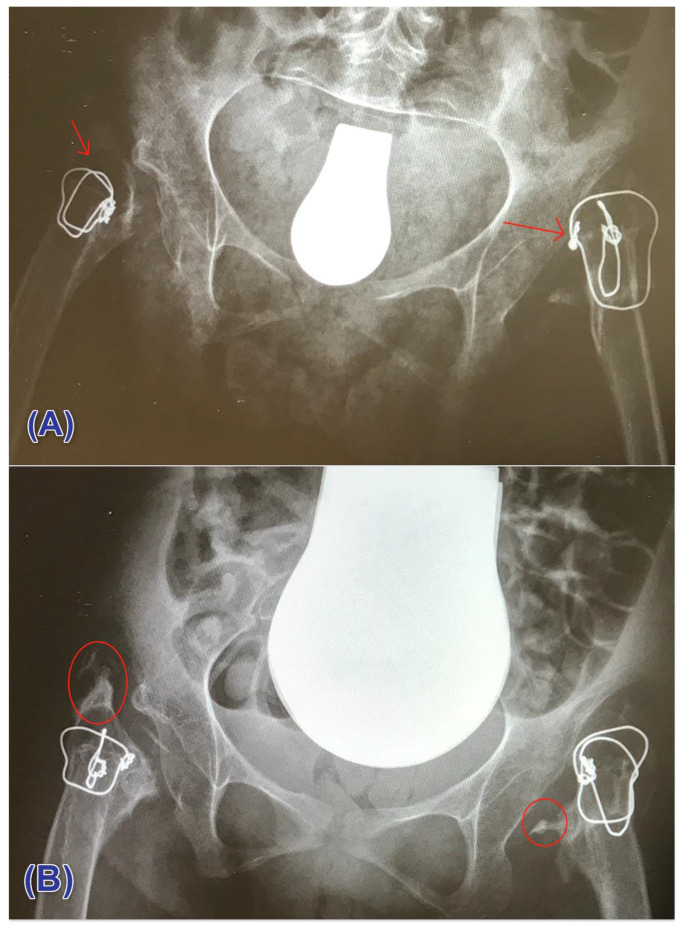
Postoperative radiograph showing (**A**) no ossification (at the site of the arrow) (**B**) heterotopic ossification (encircled).

Noteworthy, no patient in both groups had ossification grade IV. A radiographic demonstration of cases that had no heterotopic ossification and those who had ossification is illustrated in (Figure 4A,B).

### 3.4. Postoperative Complications

Overall, patients in the FHR group had less severe complications compared to patients in the FCP group (Table 3). In the FHR group, 28% had no complications (grade 0), 28% had grade II, 4% had grade II, 20% had grade III, and none had grade IV complications. Within this group, we divided patients based on their history of preoperative traction use. Thirteen patients had preoperative traction prior to being included in this study, and this group had more complications compared to those with no prior traction. Also, only one patient who underwent proximal FHR had a re-resection surgery.

On the other hand, patients in the FCP group had more complications: 13.3%, 33.3%, 10%, 16.7%, and 3.3% had grade 0, I, II, III, and IV complications, respectively. None of the patients in both groups died after the procedure (grade V).

## 4. Discussion

This study is the first, that underlines the importance of traction weight in the postoperative management of salvage hip procedures in patients with CP. To Date, there is no conclusive evidence to support the use of a certain salvage surgical approach over other methods in CP patients with painful hip or sitting problems due to neurogenic hip dislocation or subluxation. Despite the presence of many procedures in this matter, FHR is considered the most commonly used surgical option in this patient population. FHR was first introduced by Castle and Schneider [15], and therefore it is frequently referred to as the “Castle procedure”. This procedure includes proximal femoral head resection with inter-positional arthroplasty. Since this operation has been noted to be associated with frequent postoperative complications, a modification of this method was implemented via the use of capping over the resected femur head through a procedure called femoral head cap plastic surgery or FCP. This procedure has been minimally investigated in the literature. Therefore, we conducted this study to compare the postoperative outcomes of FHR and FCP.

In our study, 15 patients (25 hips) were included in the FHR group, and 23 patients (30 hips) were included in the FCP group. Baseline demographic and clinical data were similar between both groups in terms of age, gender, and GMFCS grading. However, the mean follow-up period of patients in the FHR group (32.25 months) was higher than that of the FCP group (24.6 months).

Although our study did not primarily focus on pain relief or posture outcomes following FHR or FCP, recent evidence shows that proximal FHR is effective in relieving pain following the procedure. In a recent systematic review of the different surgical options for patients with non-ambulatory CP and hip dislocation/subluxation [9], it was highlighted that proximal FHR is a reliable method in achieving pain relief, with 58.8–90% of patients experiencing pain relief after the procedure [10,15,16,17,18,19,20,21,22,23,24,25]. Despite this, FHR is associated with frequent and unique complications in the long-term, the most important of which are heterotopic ossification and telescoping. Telescoping was defined as proximal femoral migration in the literature. Heterotopic ossification is known as the most frequently encountered postoperative complication following FHR, with a prevalence rate ranging from 25 to 100% of patients [10,15,16,17,18,19,20,21,22,23,24,25]. Consistently, our study highlights similar findings where 80% of patients in the FHR group had heterotopic ossification (of grades I, II, and III), which is similar to that of the FCP group (83.3%), indicating no superiority of one method over the other regarding this outcome. Of note, none of the patients in both groups had hip ankylosis (heterotopic ossification grade IV).

Due to the high rate of heterotopic ossification in CP patients undergoing proximal FHR, surgeons made several attempts to prevent it from occurring. For example, Egermann et al. [20] modified the original FHR method by using the resected femur head like a cap secured at the resection level. In their cohort, a total of 31 patients (43 hips) were divided based on the surgical approach: 29 hips underwent FHR with capping, and 14 hips underwent FHR alone. Surprisingly, the authors noted that FHR with capping was associated with a remarkable reduction in the rate of heterotopic ossification compared to FHR alone (44.8% vs. 64.3%), respectively. Our finding does not go in line with their results, as we noted similar rates of heterotopic ossification between FHR and FCP. This difference is noted in all ossification grades. This difference could be explained by the fact the mean follow-up of patients in the FHR and capping group in their study was much shorter than that of the FHR alone group (2.2 vs. 3.7 years), respectively. This could explain why a fewer rate of heterotopic ossification was noted in the FHR and capping group. In addition, our experience shows that patients after FCR often develop massive heterotopic ossification with complete destruction of the femoral head. Due to the short follow-up, this was obviously not seen in the study performed by Egermann et al. [20]. Another explanation is the difference between the techniques used in their study and ours.

Another major complication of salvage surgical procedure is telescoping or proximal femoral migration since it interferes with sitting tolerance and can result in skin ulcers. In our study, the rate of telescoping in the proximal FHR group was 18.68%, which is similar to that reported in the literature. In the study of Castle and Schneider [15], 21.4% of patients who underwent FHR experienced telescoping. In the literature, the rate of postoperative telescoping following FHR ranges from 4% to 25% [22,26]. Comparing the frequency of telescoping in different surgical options is minimally investigated in the literature. In our study, telescoping was higher in the FCP group compared to the FHR group (18.68% vs. 31.99%); however, this difference did not reach statistical significance. This could be related to the lack of sufficient power to detect a statistically significant difference between both groups, and therefore, future studies are encouraged to include a higher sample size to confirm this observation. Meanwhile, the study of Egermann et al. [20] is the only study to compare the rate of telescoping in patients who underwent FHR alone and FHR with capping. The authors noted similar proximal femoral migration between both groups (100% vs. 100%) for the FHR alone group and FHR and capping group. The authors further did subgroup analysis based on the level of femoral migration and similar rates were noted at different subgroups. For instance, telescoping at the level of the acetabulum occurred in 64.3% and 65.5% of patients in the FHR alone and FHR and capping groups, respectively.

In our study, we examined whether telescoping was associated with either spasmolytic use, traction weight, or traction duration in the overall population (regardless of the surgical method). Of great importance, we noted that only traction weight was significantly associated with telescoping; patients who had traction of more than 2 kgs were less likely to develop telescoping compared to those who had traction of fewer than 2 kgs. This finding is novel in the current literature. However, this finding needs further confirmation by future studies since only 3 patients were included in the traction group of fewer than 2 kgs. As for spasmolytic use and traction duration, no significant association was noted with telescoping. This could be due to the small number of included patients in these analyses.

In terms of overall postoperative complications (grades I, II, III, and IV), similar rates were observed between FHR and FCP groups. None of the patients died in both group (grade V = 0). However, 4 cases in the FCP group had re-resection, while only one patient in the FHR group had re-resection.

Pain is a frequent complication in CP patients, with the hip joint being one of the most common sites where pain is reported [27]. Although it is still unclear what are the exact causes of hip pain in CP, some correlated factors are reported in the literature. Out of these factors, hip displacement or dislocation is the most prevalent [28]. Therefore, the goal in non-ambulatory CP patients is integration, participation, positioning and transfer capability, and reduction of pain caused by malposition or contractures.

Hip surveillance in children with CP is of critical importance in determining the most appropriate management approach, as reported previously in the literature [29]. Also, patients presenting with GMFCS IV or V are known to be associated with the highest rate of hip dislocation [29]. The outcomes of current hip reconstructive surgeries are hypothesized to be favorable in patients with GMFCS IV or V, especially if these surgeries are carried out in this patient population early during the disease course (early recognition) and not only be considered when patients present with pain [30]. That being said, it is of great importance to mention that this was not applicable in our patient population because CP patients did not undergo initial surveillance prior to hip surgery, and therefore, the outcomes of the only two surgical procedures (FHR and FCP) conducted at our institution were reported in this manuscript, and that is also why we could not compare between reconstructive and palliative surgery.

Our study provides novel and helpful insights into the management of hip dislocation in patients with CP and hip dislocation. Confounding factors were reduced with regard to a monocentric analysis and the definition of inclusion and exclusion criteria. That being said, we have a number of limitations.

More than half of the eligible population was not included in the analysis, majorly because of losses to follow-up. The small number of included patients in our study further limits the generalizability of our study while accounting, to a limited extent, for the insignificance of our findings. Furthermore, the difference in follow-up periods between both groups could potentially account for higher complication rates in the group with the longer follow-up period. Therefore, future studies are recommended to have a prospective design with a standardized follow-up period for both groups. Although we noted no significant change in telescoping according to the status of spasmolytic use, data regarding their dosages, frequency, and time since medication initiation were unavailable, which could potentially explain why no significant change was noted with spasmolytic use. Of note, our primary outcome was not to investigate the efficacy of FHR or FCP on pain or sitting measures, and therefore, no data regarding these outcomes are reported. Also, some patients could have undergone prophylactic radiation to reduce the risk/incidence of heterotopic ossification [31], which could affect our results. However, none of these data were available for analysis.

## 5. Conclusions

The postoperative outcomes of FHR and FCP are similar in terms of telescoping, heterotopic ossification, and complications. Although telescoping was encountered more in the FCP group, no significant difference from the FHR group was noted. In this context, we noted that the weight of traction could reduce the development of telescoping. Therefore, future studies are still warranted to confirm this observation. Based on the several limitations in our study, we recommend conducting prospective cohort studies to give more validity to the outcomes of FHR and FCP use in patients with CP.

## Figures and Tables

**Table 1:** Baseline characteristics of included patients with ICP.

Variable	Sub-Category	FHR (N = 15)	FCP (N = 23)
**Age (years); mean (SD)**			
		25.93	26.74
**Gender; N (%)**			
	Male	9 (60%)	14 (60.9%)
	Female	6 (40%)	9 (39.1%)
**Operated side; N (%)**			
	Unilateral	7 (46.7%)	15 (65.2%)
	Bilateral	8 (53.3%)	8 (34.8%)
**GMFCS; N (%)**			
	Grade IV	2 (13.3%)	5 (21.7%)
	Grade V	13 (86.7%)	18 (78.3%)
**Follow-up (months); mean (SD)**			
		32.25	24.6

SD: Standard Deviation; N: Number; FHR: Femur Head Resection; FCP: Femoral head Cap Plastic surgery.

**Table 2:** The incidence of heterotopic ossification in FHR and FCP groups.

Grade	FHR (N = 25)		FCP (N = 30)	
	N	%	N	%
0	5	20.00%	5	16.70%
I	9	36%	6	20%
II	9	36%	16	53.30%
III	2	8.00%	3	10%
IV	0	0%	0	0%

Data are provided for hips and not individual patients since some cases had bilateral surgery. N: Number; FHR: Femur Head Resection; FCP: Femoral head Cap Plastic surgery.

**Table 3:** The incidence of different complication grades in both the FHR and FCP groups.

Grade	FHR (N = 25)		FCP (N = 30)	
	N	%	N	%
Grade I	7	28%	4	13.30%
Grade II	7	28%	10	33.30%
Grade III	1	4%	3	10%
Grade IV	5	20%	5	16.70%
Grade V	0	0%	1	3.30%

FHR: Femur Head Resection; FCP: Femoral head Cap Plastic surgery; N: Number.

## Data Availability

All of the data analyzed in this manuscript can be provided upon request by contacting the corresponding author.

## Supplementary Materials

The following are available online at https://www.mdpi.com/article/10.3390/children8121105/s1, Supplementary Figure S1. Measurement of the distance between the articular surface and the femur after surgery, Supplementary Figure S2. A postoperative radiograph showing no telescoping. (A) hip joint; (B) the distance between A and C; (C) the femur, Supplementary Figure S3. A radiograph showing telescoping at follow-up. (A) hip joint; (B) the distance between A and C; (C) the femur, Supplementary Figure S4. The correlation between telescoping and traction weight, Supplementary Figure S5. The correlation between traction duration and telescoping. Supplementary Table S1. Heterotopic ossification classification, Supplementary Table S2. Classification of complications using the modified Clavien-Dindo-Sink classification system.
