# Supplementary material for: Radiological Outcomes of Femoral Head Resection in Patients with Cerebral Palsy: A Retrospective Comparative Study of Two Surgical Procedures
Source: Children (Basel). 2021 Dec 1;8(12):1105. doi: 10.3390/children8121105 (PMC8700049; doi:10.3390/children8121105)
Supplement: Supplementary file 1 [file children-08-01105-s001.zip › children-1451168-supplementary.pdf]

Supplementary File

Supplementary Table S1. Heterotopic ossification classification

Grade	Radiographic evidence
0	No evidence of the bone island
I	Islands of bone within the soft tissue around the hip
II	Bone spurs from the pelvis or proximal end of the femur, leaving at least one centimeter between opposing surfaces
III	Bone spurs from the pelvis or proximal end of the femur, reducing the space between opposing bone surfaces to less than one centimeter
IV	Apparent bone ankylosis of the hip

Supplementary Table S2. Classification of complications using the Clavien-Dindo-Sink classification system

Degree of Complication	Examples
I: (Any deviation from normal postoperative course, no intervention required)	No postoperative adverse events
II: (Pharmaceuticals, blood transfusion, parenteral nutrition)	Pain medication adjustment
	oral antibiotic therapy for wound infections, postoperatively
III: (Surgical, endoscopic or radiological intervention necessary).<br>IIIa = without general anesthesia;<br>IIIb = with general anesthesia	Transfusion
	Urinary tract infection with antibiotic use
	Wound infection with intravenous antibiotics
IV: (Life-threatening intensive medical treatment necessary)<br>IVa= single organ failure<br>IVb = multiorgan failure	Re-resection for pain/cerclage fracture
	Wound dressing because of wound infection
V: (Dead)	Intensive care unit due to respiratory distress, postoperatively

Supplementary Figures

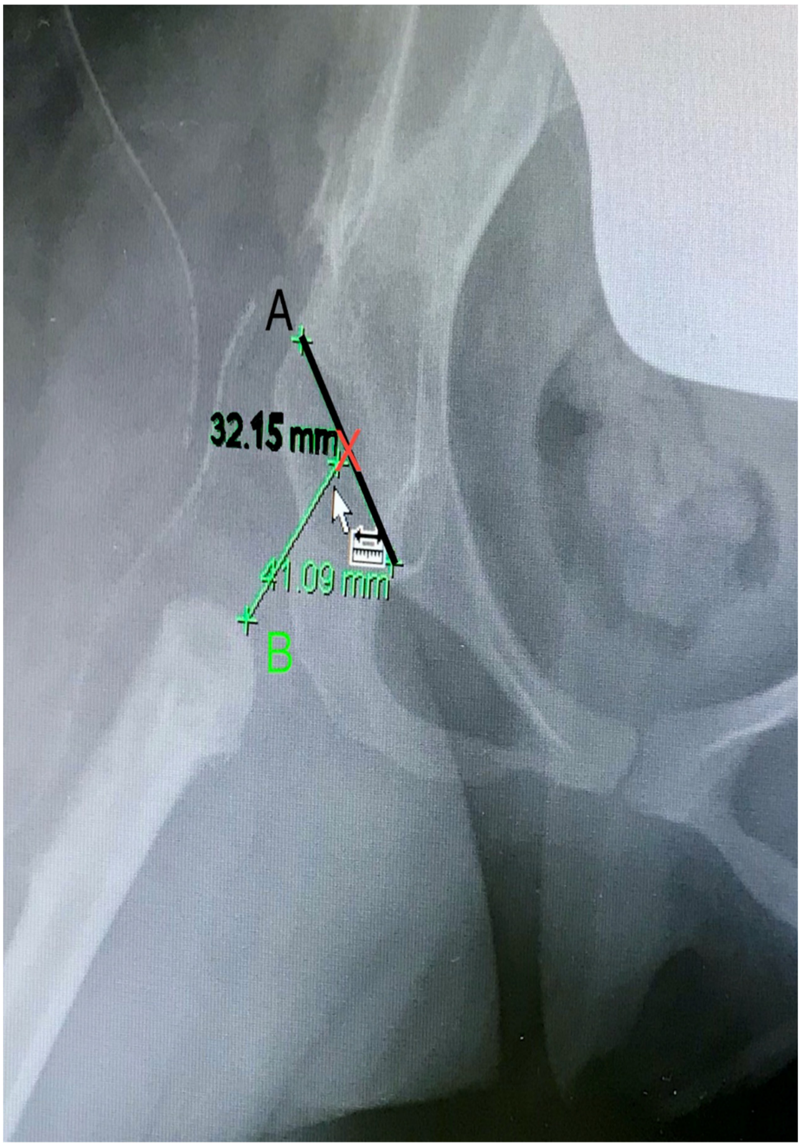

Supplementary Figure S1. Measurement of the distance between the articular surface and the femur after surgery

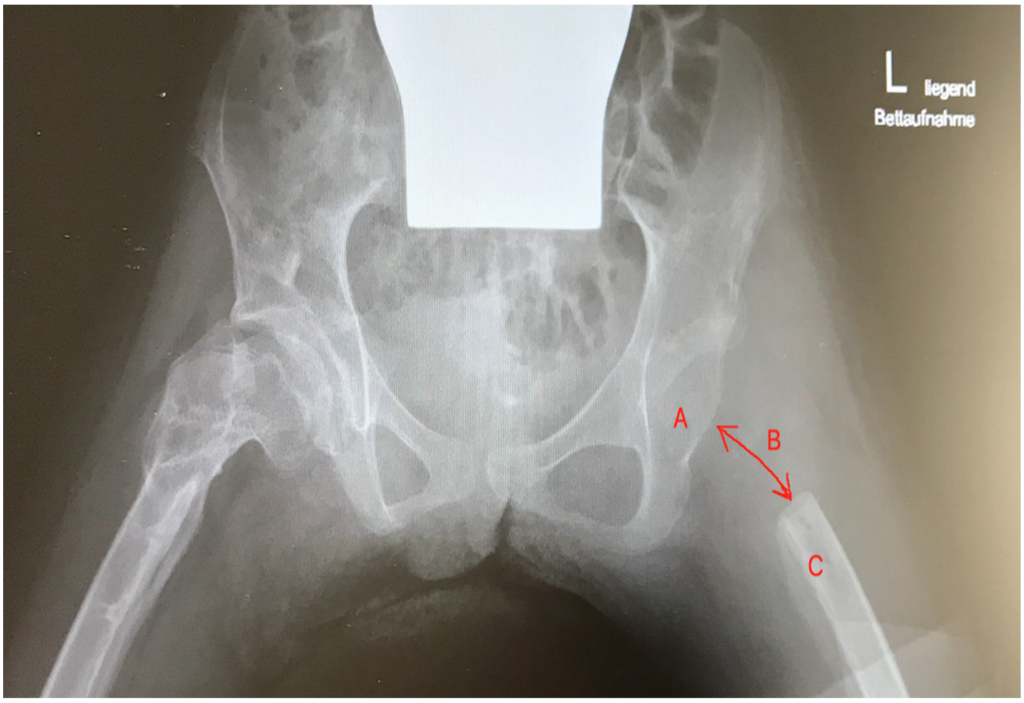

Supplementary Figure S2. A postoperative radiograph showing no telescoping. (A) hip joint; (B) the distance between A and C; (C) the femur

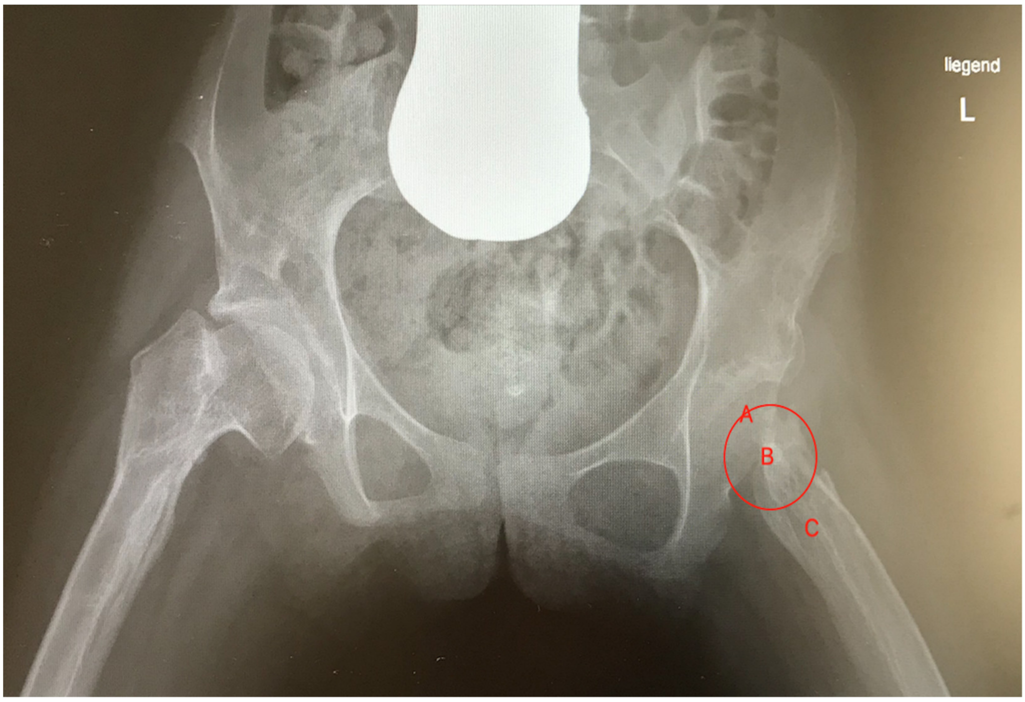

Supplementary Figure S3. A radiograph showing telescoping at follow-up. (A) hip joint; (B) the distance between A and C; (C) the femur

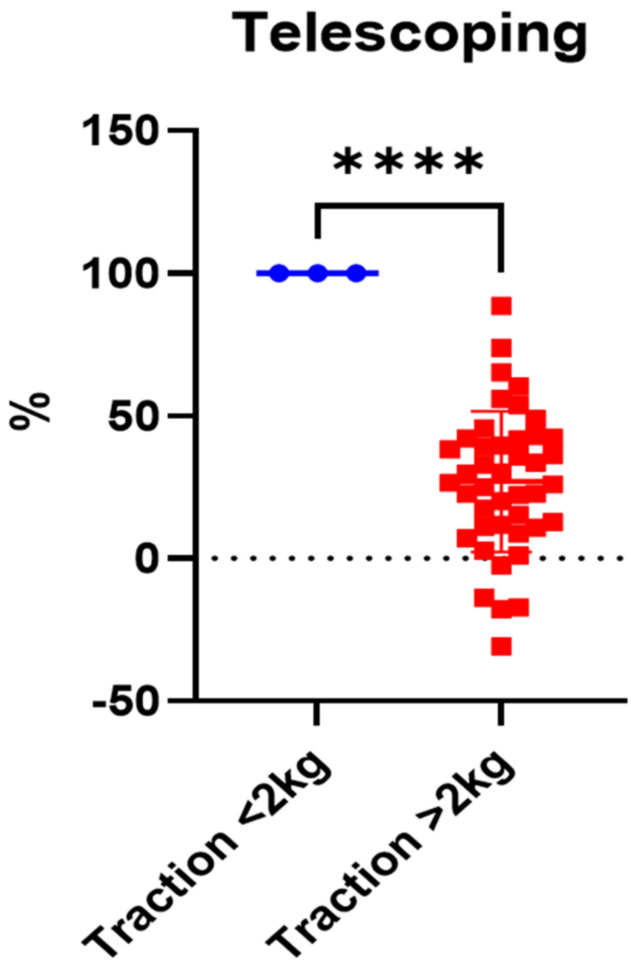

Supplementary Figure S4. The correlation between telescoping and traction weight

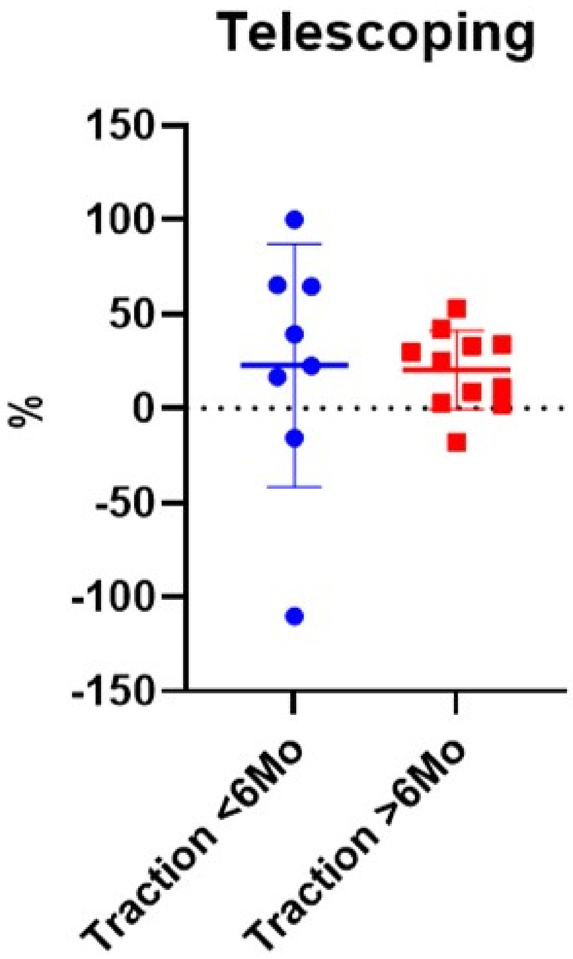

Supplementary Figure S5. The correlation between traction duration and telescoping
